# COVID-19 in Wuhan, China: Pressing Realities and City Management

**DOI:** 10.3389/fpubh.2020.596913

**Published:** 2021-02-17

**Authors:** Rita Yi Man Li, Xiao-Guang Yue, M. James C. Crabbe

**Affiliations:** ^1^Department of Economics and Finance/Sustainable Real Estate Research Center, Hong Kong Shue Yan University, North Point, Hong Kong; ^2^School of Sciences, European University of Cyprus, Engomi, Cyprus; ^3^Wolfson College, Oxford University, Oxford, United Kingdom; ^4^Institute of Biomedical and Environmental Science and Technology, University of Bedfordshire, Luton, United Kingdom; ^5^School of Life Sciences, Shanxi University, Taiyuan, China

**Keywords:** COVID-19, city management, Wuhan, health policy, socialism

## Introduction

To most economists around the World, Covid-19 has provided an objective lesson in market failure (1). In the absence of complete information and sometimes even fake news (2), nobody knew what kind of pandemic it was at the beginning. Yet, there were 32,583 patients with laboratory-confirmed Covid-19 in Wuhan between December 8, 2019, and 8 March 8, 2020 (3). The pandemic crippled and continues to cripple many health systems and has created unprecedented pressure on the psychological and physical aspects of millions of people's lives around the world. Over 200 countries and territories suffer from an acute shortage of medical personnel and medical equipment (4).

The responses of different countries to Covid-19 has involved a range of measures that reflect national values, politics, and variations in scientific advice provided by local experts. Political considerations have often become more important than science (5). The Covid-19 outbreak in Wuhan was one of the most serious cases amongst all cities in the world, yet Wuhan managed and gain control of this pandemic.

Health care systems and policies are important aspects that affect the control of infectious diseases like Covid-19. In China, 98% of primary health-care is complemented by traditional Chinese medicine (TCM) with allopathic approaches (6). Previous research has found that the cure rate increased by 33% among mild cases after adopting TCM with allopathic approaches. The hospital stay of severe patients with TCM's and nucleic acid turning negative was shortened by over 2 days (7). Prior to Covid-19, the government's Healthy China 2030 plan was already addressing chronic diseases in the aging population by raising healthcare expenditure (6). This is in sharp contrast to other countries with aging population problems such as Italy, where the government cut the healthcare budget substantially after the economic downturn. Hospital bed allocation went down from a maximum of four for every thousand inhabitants to a maximum of 3.7 (8), despite the fact that 23.1% of the Italian population were aged 65 years and older in 2020 (9). Likewise, the post-2008 financial crisis in Spain forced severe cuts to healthcare costs, which caused pressure on the system when there was an increase in demand for healthcare services. These measures particularly affected the elderly and disabled who are more vulnerable to Covid-19. Healthcare costs become underfunded at the level of 6.4% of GDP (10).

Apart from scientific evidence on the effectiveness of TCM in curing covid, financial expenditures on health care is an important distal factor that helped Wuhan overcome Covid-19 quickly. In the following sections of this paper, we review the three city management stages adopted in Wuhan, to study proximal causes of success in combating the virus: (1) strong government intervention early in the outbreak; (2) the city lockdown; and, (3) the use of digital measures, such as a health code, when the city reopened.

## Three Stages of COVID-19 in Wuhan

### Stage 1: December 2019 to January 22, 2020 (Early Covid Outbreak in the Absence of Strong Government Intervention)

In December 2019, some cases of “pneumonia of unknown origin” were reported in Wuhan. Chinese health authorities confirmed that this cluster was associated with a coronavirus (11). Most activities took place as normal. By Jan 2, 2020, 41 patients admitted to hospital were identified as having a laboratory-confirmed 2019-nCoV infection. Altogether 27 of these 41 patients had been to the Huanan seafood market between December 8, 2019, and January 2, 2020, and it was then closed on January 1, 2020. One family cluster was found. With the Chunyuan, a massive population flow for the Chinese New Year started on January 10, 2020, and the increase in movement between provinces escalated the transmission of Covid-19 (from January 10 the numbers increased from around 100 cases to 400 cases on January 19, alone). The National Health Commission confirmed human-to-human transmission of Covid-19 on January 20, 2020 (12). The Chinese Health Authorities notified the World Health Organization (WHO) on December 31, 2019, and stepped up border surveillance, prompted by Hong Kong, Macau, and Taiwan (13, 14). At first, a lack of information prevented authorities in China from implementing effective measures, but it was soon established that early warning and traffic restrictions are important measures in controlling the early onset of coronavirus and reducing the spread of the virus to other places.

### Stage 2: January 23, 2020, to Mid-March 2020 (Close City Management Period)

After having over 1,000 covid cases confirmed on January 22 2020 alone, the Wuhan Municipal Government adopted “close city” management on January 23, 2020, meaning nobody could move to Wuhan, and everyone inside the city had to quarantine. Locals began to store food, medicine, and protective equipment (15). Yet, Wuhan had over a 100 cases, which went up by 100 per day on average (as compared to the previous day) from January 10 onwards. From January 22 to February 5 2020, a thousand covid new cases were diagnosed daily on average, reaching the peak of 2,000 1 day on February 1, 2020 (3). The insurmountable number of newly infected patients overloaded the existing 83 hospitals in Wuhan (16) and drove Wuhan to designate several hospitals with 23,532 beds, which admitted only covid patients. New temporary hospitals such as the Huoshenshan Hospital and the Leishenshan Hospital were built to provide an additional 2,600 beds but their capability to treat patients was limited compared to the increase in patients (12) (Appendix in Supplementary Tables 1, 2).

This initiative involved 140 medical volunteers from Jiangsu, who arrived in Wuhan on January 23, 2020 (17) and 136 medical volunteers from Shanghai Medical Team Huashan Hospital, Ruijin Hospital, Xinhua Hospital, Chest Hospital, Renji Hospital in Shanghai arrived on January 25, 2020, to relieve the pressure of medical staff in Wuhan (18). Subsequently, over 32,572 volunteer health personnel from other provinces gradually came to Wuhan in this period (19). All residential areas were closed and people from outside could not enter. People sent messages to community WeChat groups or QQ groups (online communities) for the purchase of necessities. Community staff bought these for them or they received free food from the government (source: author's WeChat group in Wuhan). Health policies were tightened quickly in February:

- Non-residents were forbidden to enter small districts on February, 14.
- Each household sent one person every 3 days to purchase daily necessities on February, 16.
- Residents had to measure body temperature twice and sent those that exceeded 37.3°C to medical institutions from February, 17 (15).

The effective reproduction number of Covid-19 kept above 3.0 before January 26 and dropped below 1.0 after February 6, and was lower than 0.3 after March 1 (3). March marked the early success of all the measures in combatting Covid-19.

### Stage 3: Late March to August 2020 (Recovery After Close City Management)

**Figure 1:**
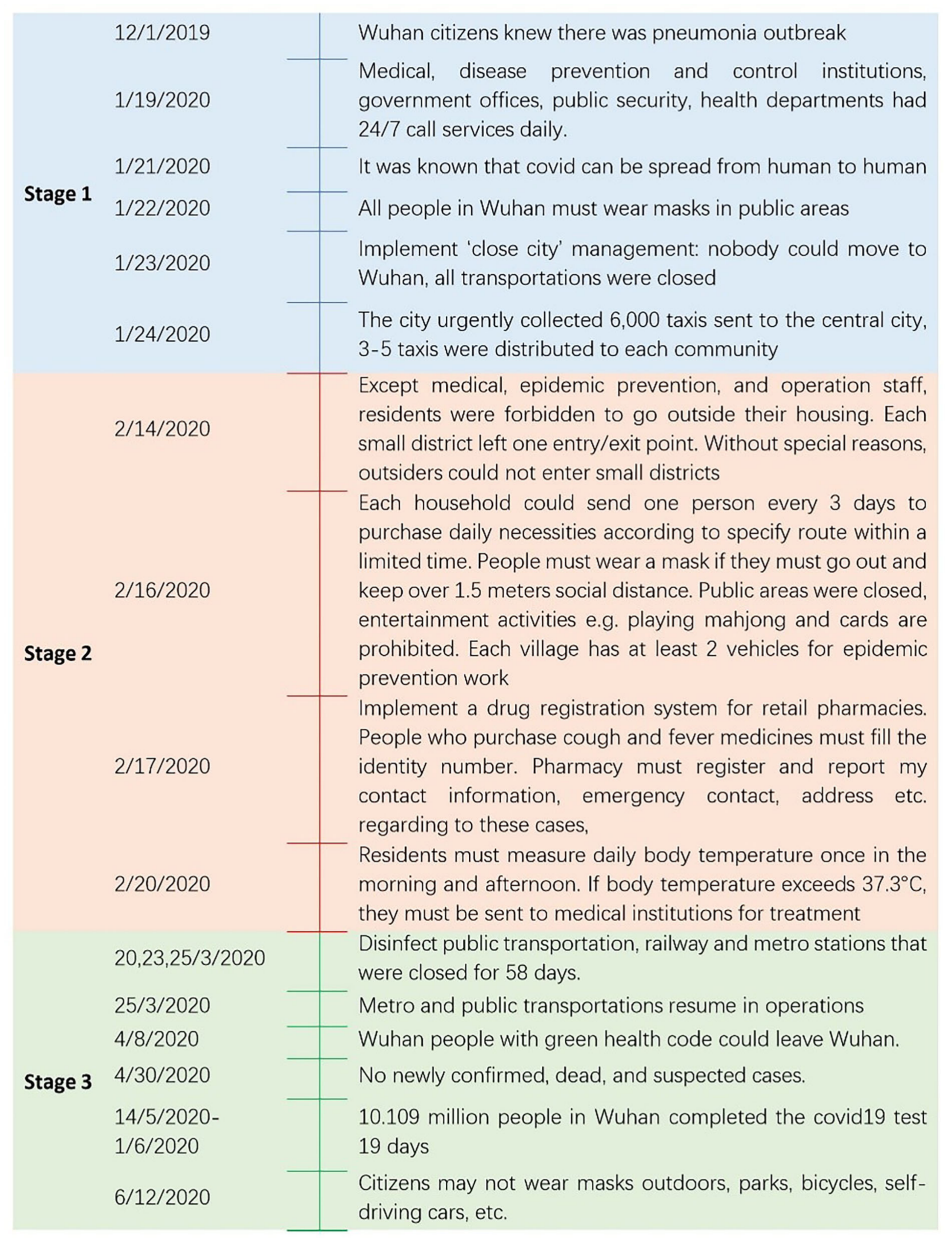
Three stages of covid-19 measurement (15, 20, 22, 23).

In late March, public transportation systems resumed services. Since April 30, 2020, there have been no further newly confirmed, dead, or suspected covid cases. From May 14 to June 1, covid test institutions operated 24-h a day, 10.109 million people in Wuhan completed a Covid-19 test within 19 days. Sixty-three medical institutions, 1,451 testing personnel, and 701 testing equipment were involved. A “Red code” was issued for Covid-19 patients in the health app linked with WeChat and Alipay. If the patient was cured, discharged, and had no recurring symptoms after 14 days of isolation, they received a “yellow code.” After returning home for 14 days, this rating was converted to a “green code.” People with fever and any people closely connected to confirmed covid patients were assigned “yellow codes.” They were given “green codes” after 14 days of quarantine and after they had passed a covid test (15, 20, 21). The three stages of Covid-19 measurement and responses are shown in Figure 1.

## Discussion and Conclusions

### A Choice Between Combating the Virus and Human Rights

To reduce the spread of Covid-19 and contain any spread of the infectious disease in the future, early response is essential (24). Most of the policy decisions discussed above were made by government officials who made prompt policies and implemented them quickly, but citizens had no involvement or say in the process. Covid-19 calls for a period of lockdown (25) which successfully prevents the spread of the virus, however, this fails to address human rights (e.g., they cannot go outside easily) and it also amplifies health inequalities (26, 27). Many people from western and other societies consider not wearing masks as a kind of human freedom, however, not wearing masks cannot prevent the spread of Covid-19. Many worried that a resurgence of the disease will happen after the country lifts the strictest control measures, although this is not supported by data as per Figure 1 and health records up to August 2020. The Chinese government implemented extraordinary labor-intensive, large-scale measures (21) like closing Wuhan city in February 2020 and giving 10.109 million people a Covid-19 test over 19 days. This is perhaps “mission impossible” anywhere else, given the requirements and budgets that are required and the fact that it may or may not test human rights.

A health code system that utilizes Covid-19 records in Alipay and WeChat was linked with the personal data of all mainland Chinese citizens, including bank accounts and debt records. This approach might not be impossible overseas, due to concerns about the freedom and privacy of individuals. The collection of 6,000 taxis within a day, for moving materials, personnel, and transporting patients is possible in China owing to its socialist political structure. It could be said that this goes against private ownership (a kind of the human rights). None of these actions could happen easily in countries in the west, yet, it was also because of all these controversial non-therapeutic measures that Covid-19 was contained and controlled in a short time.

### TCM, Budget, and Preparation for the Future

The use of TCM together with western medicine and an appropriate budget for healthcare are important distal causes in combating Covid-19. Budget cuts in other countries after the financial crisis has led to a serious healthcare problem, particularly when these systems are faced with an unexpected Covid-19 pandemic. Finally, the outbreak of SARS in 2003 followed by Covid-19 indicates that it is likely that other coronavirus diseases may happen again in the future. Given the high population density in urban areas, large-scale public venues in urban areas should allow open building concepts that allow the existing building infrastructures to be converted to medical emergency centers. Local emergency action plans including sewage and ventilation prerequisites and procedures for renovation should be recorded and revised according to our experience in Wuhan and other places (12). With appropriate leadership and politics, these measures might be adapted to other cities across the globe.

## Author Contributions

RL and XY wrote and collected the data. MJCC revised and edited the whole paper. All authors contributed to the article and approved the submitted version.

## Conflict of Interest

The authors declare that the research was conducted in the absence of any commercial or financial relationships that could be construed as a potential conflict of interest.
