# Supplementary material for: COVID-19 in Wuhan, China: Pressing Realities and City Management
Source: Front Public Health. 2021 Feb 17;8:596913. doi: 10.3389/fpubh.2020.596913 (PMC7925403; doi:10.3389/fpubh.2020.596913)
Supplement: Supplementary file 1 [file Table_1.docx]

| **Appendix**  **Table 1** Designated, newly built, temporary and Fangcang shelter hospitals in Wuhan from 22 January 2020 to 5 February 2020 (12) (15) (28). | | | |
| --- | --- | --- | --- |
| **Type of hospital** | **Designated hospitals** | **Newly built temporary hospitals** | **Fangcang shelter hospitals** |
| Approaches | Over 40 designate several hospitals to admit covid patients. | Wuhan Huoshenshan Hospital and Leishenshan Hospital were built to meet the requirements of respiratory infectious disease hospitals | Requisition of over 20 public venues and converedt them to healthcare facilities |
| Date of opening | 22 January 2020 | 23 January 2020 | 5 February 2020 |
| Number of beds | 23 532 | 2600 (Huoshenshan 1000, Leishenshan 1600) | 16 000 |
| Merit | Equipped with the necessary medical equipment to provide sufficient care to serious covid patients. | The hospitals were in accordance with covid control standards. | Rapid construction, cheap and large scale. |
| Shortcoming | High risk of nosocomial infection, and it difficult to provide different levels of care. | Time-consuming and costly construction, limited hospital beds. | Public venues did not meet the requirements of hospitals and comfortable living conditions in general. |
| Applicable when | Covid patients in critical situation | Covid patients reached or exceeded the designated hospitals’ capacity | Covid case confirmed but the patients are not in severe conditions |

Table 2 Newly refurbished Fangcang hospitals in Wuhan (12)

| **Name** | **District** | **Original function** | **Area (10 000 m^2^)** | **Distance between the nearest neighbourhood and the hospital (m)** | **Nearest designated hospital and distance (km)** |
| --- | --- | --- | --- | --- | --- |
| Hanyang Fangcang Shelter Hospital | Hanyang | Wuhan International Expo Center | 18 | 883 | Wuhan Traditional Chinese Medicine Hospital (2.9) |
| Jianghan Fangcang Shelter Hospital | Jianghan | Wuhan International Convention and Exhibition Center | 12.7 | 162 | Wuhan Fourth Hospital (1.6) |
| Wuhan Living Room Fangcang Shelter Hospital | Dongxihu | Wuhan Living Room | 12.5 | 959 | Wuhan Jinyintan Hospital (0.8) |
| Dahuashan Fangcang Shelter Hospital | Jiangxia | Dahuashan Outdoor Sports Center | 7.95 | 577 | The First People’s Hospital of Jiangxia District (3.1) |
| Wuhan National Fitness Center Fangcang Shelter Hospital | Jiang’an | Wuhan National Fitness Center | 6.45 | 473 | Wuhan Central Hospital (2.3) |
| Optics Valley Sunsea Fangcang Shelter Hospital | Hongshan | Wuhan Meilian Group’s former Rihai Industrial Park plant | 5.4 | 319 | East Hospital of People’s Hospital of Wuhan University (5.3) |
| Wuhan Sports Center Stadium Fangcang Shelter Hospital | Caidian | Wuhan Sports Center Stadium | 5.07 | 632 | West Hospital of Union Hospital, TJMU (1.0) |
| Shipailing Fangcang Shelter Hospital | Hongshan | Shipailing Senior Vocational High School | 4.6 | 149 | Tianyou Hospital (1.5) |
| Changjiang New Town Fangcang Shelter Hospital | Jiang’an | Hongqiao Industrial Park | 2.75 | 570 | Wuhan Jinyintan Hospital (2.2) |
| Wuchang Fangcang Shelter Hospital | Wuchang | Hongshan Stadium | 2 | 363 | Wuhan Seventh Hospital (0.5) |
| Caidian Fangcang Shelter Hospital | Caidian | Yangtze River Media Zhiyin Practice Training Base | 1.79 | 211 | Caidian District People’s Hospital of Wuhan (6.8) |
| Wuhan Stadium Fangcang Shelter Hospital | Qiaokou | Wuhan Stadium | 1.45 | 133 | Wuhan Fourth Hospital (1.3) |
| Optics Valley Fangcang Shelter Hospital | Hongshan | Optics Valley Convention and Exhibition Center | 1 | 742 | Tongji Hospital (4.4) |
| Huangpi Stadium Fangcang Shelter Hospital | Huangpi | Huangpi Stadium | 0.74 | 550 | Huangpi District People’s Hospital (2.1) |
| Dunkou Stadium Fangcang Shelter Hospital | Hannan | Kirin Logistics Plant | 0.63 | 635 | West Hospital of Union Hospital, TJMU (4.6) |
| Qingshan Fangcang Shelter Hospital | Qingshan | WISCO Sports Center | 0.2 | 378 | Wuhan Ninth Hospital (1.5) |

**REFERENCE**

28. Hu Y. *Graphics: Does Wuhan Have Enough Hospital Beds for Coronavirus Patients?* (2020). Available online at: https://news.cgtn.com/news/2020-02-18/Coronavirus-Does-Wuhan-have-enough-hospital-beds--ObxZUAhuJG/index.html
